# Feasibility of a Three-Dimensional Porous Uncalcined and Unsintered Hydroxyapatite/poly-d/l-lactide Composite as a Regenerative Biomaterial in Maxillofacial Surgery

**DOI:** 10.3390/ma11102047

**Published:** 2018-10-20

**Authors:** Yunpeng Bai, Takahiro Kanno, Hiroto Tatsumi, Kenichi Miyamoto, Jingjing Sha, Katsumi Hideshima, Yumi Matsuzaki

**Affiliations:** 1 Department of Oral and Maxillofacial Surgery, Shimane University Faculty of Medicine, 89-1 Enya-Cho, Izumo, Shimane 693-8501, Japan; xyywq@126.com (Y.B.); tatsumi@med.shimane-u.ac.jp (H.T.); jsswjbnjw@gmail.com (J.S.); hideg@med.shimane-u.ac.jp (K.H.); 2 Division of Oral and Maxillofacial Surgery, Oki Hospital, 355 Johokumachi, Okinoshima-Cho, Oki-Gun, Shimane 685-0016, Japan; 3 Department of Cancer Biology, Shimane University Faculty of Medicine, 89-1 Enya-Cho, Izumo, Shimane 693-8501, Japan; miyaken@med.shimane-u.ac.jp (K.M.); matsuzak@med.shimane-u.ac.jp (Y.M.)

**Keywords:** uncalcined and unsintered hydroxyapatite/poly-d/l-lactide, bioactive resorbable plate, cell proliferation, cell differentiation, osteoconductivity

## Abstract

This study evaluated the feasibility of a novel three-dimensional (3D) porous composite of uncalcined and unsintered hydroxyapatite (u-HA) and poly-d/l-lactide (PDLLA) (3D-HA/PDLLA) for the bony regenerative biomaterial in maxillofacial surgery, focusing on cellular activities and osteoconductivity properties in vitro and in vivo. In the in vitro study, we assessed the proliferation and ingrowth of preosteoblastic cells (MC3T3-E1 cells) in 3D-HA/PDLLA biomaterials using 3D cell culture, and the results indicated enhanced bioactive proliferation. After osteogenic differentiation of those cells on 3D-HA/PDLLA, the osteogenesis marker genes runt-related transcription factor-2 (Runx2), and Sp7 (Osterix) were upregulated. For the in vivo study, we evaluated the utility of 3D-HA/PDLLA biomaterials compared to the conventional bone substitute of beta-tricalcium phosphate (β-TCP) in rats with critical mandibular bony defects. The implantation of 3D-HA/PDLLA biomaterials resulted in enhanced bone regeneration, by inducing high osteoconductivity as well as higher β-TCP levels. Our study thus showed that the novel composite, 3D-HA/PDLLA, is an excellent bioactive/bioresorbable biomaterial for use as a cellular scaffold, both in vitro and in vivo, and has utility in bone regenerative therapy, such as for patients with irregular maxillofacial bone defects.

## 1. Introduction

The maxillofacial area is a relatively complex part of the human body, consisting of bone, cartilage, and networks of nerves and vessels [1]. Critical-size segmental bone defects in this region occur as a result of cancer resection, trauma, congenital malformations, and progressive skeletal deformity [2]. Reconstruction of maxillofacial bones is difficult due to the unique aesthetic requirements and functional demands, which include mastication and the expression of emotions [3]. Furthermore, bite force also plays a role in the reconstruction procedure. The material used to fill the bony defect should be endowed with sufficient compressive strength to sustain maxillofacial bone and bear strong masticatory pressure [4].

Several methods have been developed for the reconstructing of maxillofacial bone, including autogenous bone grafts, alloplasts, and allografts [5]. Autografts have been used to repair bone defects and usually achieve good results. Nevertheless, they require two surgical procedures, bone harvesting and implantation, which increases morbidity and mortality. Simultaneously, the volume of bone available for harvesting is limited. In the case of allografts, the disadvantages are the risk of inducing an immune response and disease transmission [6].

Due to their different limitations and characteristics, various types of synthetic bone substitutes have been developed as an alternative to bone grafts [1]. Synthetic material may provide a solution to the limited availability of autologous bone grafts and overcome the disadvantage of an induced immune response. Thus far, calcium phosphate ceramics, with their remarkable biocompatibility and osteoconductivity, have yield good clinical results [7].

Hydroxyapatite (HA) is the main component of bone mineral. It is a calcium phosphate ceramic with a macroporous structure similar to that of cancellous bone. The advantages of its use as a bone substitute are its excellent biological properties, its porous structure, good osteoconductivity, and biocompatibility, and its ability to promote the infiltration of bone, bone marrow, and blood vascular cells [8,9]. Poly-d/l-lactide (PDLLA), a polymer with a controllable degradation rate and good biocompatibility, is widely used in tissue engineering [10]. To develop better bioresorbable bone graft substitutes, HA particles have been incorporated into a PDLLA matrix to produce three-dimensional (3D)-HA/PDLLA composites along with various devices of different shapes and sizes.

Previous studies concluded that the mechanical strength and strength retention of 3D-HA/PDLLA recommend its use for the reconstruction of long bones, such as the femoral condyles [11]. However, such results have not been demonstrated in irregular bones, such as the maxillofacial region. In this study, we attempted to further clarify the unique properties of this 3D-HA/PDLLA composite as a bone regeneration biomaterial for mandibular bony defects and its influence on cellular responses. We performed in vitro studies comparing the 3D-HA/PDLLA composite with dense-HA/PDLLA and beta-tricalcium phosphate (β-TCP) in vivo. A better understanding of the 3D-HA/PDLLA composite will contribute to the development of a new generation of bioactive/bioresorbable materials, allowing for increased long-term biocompatibility in maxillofacial surgery.

## 2. Materials and Methods

### 2.1. In Vitro Experiments

#### 2.1.1. 3D-HA/PDLLA and Dense-HA/PDLLA Composite Biomaterial

Porous 3D-HA/PDLLA composite was provided by Teijin (Teijin Medical Technologies Co., Ltd., Osaka, Japan). Porosity was calculated from the apparent density: 70%; pore diameter: 40–480 μm, average of 170 μm. The pores of the 3D-HA/PDLLA composite are interconnected with an average interconnective pore diameter of 62 μm. The 3D-HA/PDLLA biomaterial was composed of 70 wt% uncalcined and unsintered HA (u-HA) and 30 wt% PDLLA matrix, with 4.1 ± 0.4 MPa compressive strength (viscosity-average molecular weight (Mv): 77 kDa; dextrorotatory-lactide acid/levorotatory-lactide acid (d/l) = 50/50 mol%) and without immediate collapse after breaking [11].

The control material, made of dense-HA/PDLLA, was also provided by Teijin. This biomaterial contains a homogeneous mixture of 3D-HA/PDLLA; however, it does not have any pores or interconnective pores inside.

**Figure 1:**
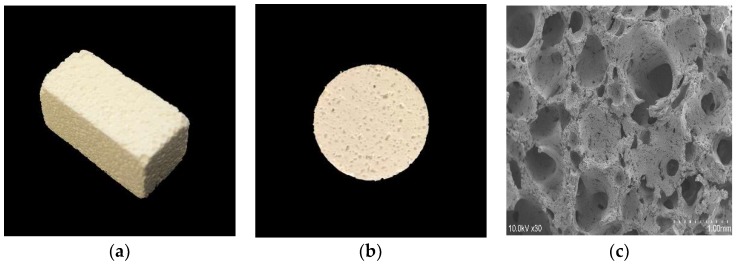
The three-dimensional porous uncalcined and unsintered hydroxyapatite and poly-d/l-lactide (3D-HA/PDLLA) biomaterial used in this research. (**a**) The size of the 3D-HA/PDLLA cellular cubic composite was 23 × 10 × 10 mm. (**b**) The size of the 3D-HA/PDLLA sheet was Φ20 × 2 mm. (**c**) Scanning electron microscope (SEM) image of 3D-HA/PDLLA scaffold. Scale bar: 1 mm.

All materials used in this study were sterilized under UV light for 30 min on each side and preincubated in α-minimal essential medium (α-MEM) in a humidified incubator with 5% CO_2_ at 37 °C overnight. The material diagram was shown in Figure 1.

#### 2.1.2. Osteoblastic Cells

The preosteoblastic cell line MC3T3-E1 was purchased from the RIKEN Cell Bank (Cell Engineering Division, RIKEN BioResource Research Center, Tokyo, Japan).

#### 2.1.3. Cell Proliferation Assay

The 3D-HA/PDLLA cellular cubes each had a border length of 1 cm. After a 24 h preincubation in α-MEM, MC3T3-E1 cells (2.0 × 10^4^ cells/cube) were injected into the center of the cubes using a 5 mL syringe with an 18-gauge needle. The cubes were cultured for 48 h in a 12-well plate containing 5 mL of α-MEM supplemented with 10% fetal bovine serum and 1% penicillin/streptomycin under 5% CO_2_ at 37 °C to investigate cell proliferation. At 0, 24, and 48 h after cell injection AlamarBlue solution (AbD Serotec Ltd., Oxford, UK) was added to the 3D culture medium at each sampling point (10 vol%), after which culture was continued for 6 h. Then, 1 mL culture samples were transferred to a 12-well plate and the fluorescence intensities were measured using a microplate reader (excitation: 570 nm; emission: 600 nm).

#### 2.1.4. Reverse Transcription (RT) Quantitative Real-Time Polymerase Chain Reaction (qRT-PCR)

3D-HA/PDLLA and dense-HA/PDLLA sheets were fabricated into Φ14 × 0.7 mm shapes and placed in a 6-well plate at a cell concentration of 4.0 × 10^5^ in 5 mL of α-MEM medium. The plates were incubated at 37 °C and 5% CO_2_ atmosphere in a humidified incubator. The culture medium was replaced every 3 days.

Total RNA was extracted from the cells cultured on the sheet samples using TRIzol reagent (Invitrogen Life Technologies, Madison, WI, USA) and 1 μg of total RNA was subjected to RT using Prime Script RT Master Mix (Takara Bio Inc., Kusatsu, Japan). qRT-PCR was performed according to the manufacturer’s protocol using SYBR Premix Ex Taq II (Takara Bio Inc., Kusatsu, Japan) on a CFX96 PCR System (Bio-Rad, Hercules, CA, USA). The concentrations of total RNA were determined by measuring the optical absorbance of the extract at 260 nm. The levels of target gene expression were normalized to that of Gapdh. Each sample was assayed in triplicate. Information on the primers is provided in Table 1. 

### 2.2. In Vivo Experiments

#### 2.2.1. Critical Bone Defection and Intraosseous Implantation

Superpore^®^ (Pentax SKM, Tokyo, Japan) was used as the β-TCP biomaterial in the in vivo study, with 60% porosity, 10 MPa compressive strength, and 100–165 μm pore diameter. The 3D-HA/PDLLA and dense-HA/PDLLA composites used were as described above. All filling materials applied in the in vivo study corresponded to complete critical bone defection, with a 4 mm diameter and 2 mm thickness.

For the mandibular bone critical defection models, 72 male Sprague Dawley (SD) rats (10 weeks old and average weight of 300–350 g; Brought from Charles River, Tokyo, Japan) were divided into four groups (18 SD rats per group): a no transplantation group as the sham group, a 3D-HA/PDLLA group, a dense-HA/PDLLA group, and a β-TCP group.

All SD rats were anesthetized by injecting pentobarbital (50 mg/kg) into the abdomen. Surgery was performed under standard aseptic conditions. A longitudinal incision approximately 1 cm in length was made through the full thickness of the mandible skin, and the muscles were separated by forceps to expose the mandible surface. At the mandible body, we created a round defect with a diameter of 4 mm that could not be self-repaired. Then, the four groups were treated separately. Defects in the sham group were not filled with any material. In the 3D-HA/PDLLA, dense-HA/PDLLA and β-TCP groups, defects were filled with 3D-HA/PDLLA, dense-HA/PDLLA, and β-TCP, respectively.

After sufficient normal saline irrigation, the wounds were closed layer by layer. The rats awoke 1–2 h after the operation and showed normal behavior and appetite. All rats were euthanized by intra-abdominal injection with an overdose of anesthetic at week 1, 2, or 4 post-operation. The mandible was harvested and soaked in 10% neutral buffered formalin for further analysis.

Surgery and treatment were performed in strict accordance with the Guidelines for Care and Use of Laboratory Animals of the Faculty of Medicine of Shimane University. The animal protocol was approved by the Animal Ethics Committee of our institution (Approval references; IZ 25–166, IZ 26–167).

#### 2.2.2. Micro-Computer Tomography (CT) Evaluation

The samples were fixed in 10% neutral buffered formalin for 7 days and then scanned by micro-CT (Inveon, Siemens, Munich, Germany), conducted by KUREHA Special Laboratory Co., Ltd. (Tokyo, Japan). The resolution of the micro-CT device was 53.645 μm/pixel, the magnification 1.864×. Radiographic analysis was conducted to assess the union of bioresorbable material and host bone, as well as the condition of the biomaterial. Three rats per group were included in the micro-CT analysis.

#### 2.2.3. Histomorphological Examination

After micro-CT, three samples from each week group were decalcified, dehydrated, and embedded in paraffin. The specimens were sectioned perpendicular to the disc and the axis of the cylinder. Hematoxylin and eosin (H&E) staining was performed for histological analysis and the stained slices were observed under a BX50 light microscope (Olympus Corp., Tokyo, Japan). The areas of ectopic bone were calculated using ImageJ software (Media Cybernetics, Inc., Rockville, MD, USA). For the 3D-HA/PDLLA and β-TCP samples, the ratio of newly formed bone volume (BV) to residual material volume (MV; BV/MV%) in the critical defection field under 20× magnification was calculated.

### 2.3. Statistical Analysis

The results of the AlamarBlue assay cell proliferation at 0, 24, and 48 h were compared using an unpaired *t*-test. The qRT-PCR results were analyzed using a one-factor ANOVA test and Tukey’s honest significant difference test. The difference in BV/MV% between the 3D-HA/PDLLA and β-TCP groups was analyzed by the Kruskal–Wallis H test. In all analyses, differences were considered significant at *p* < 0.05.

## 3. Results

### 3.1. Cell Proliferation of Cubic Composites

**Figure 2:**
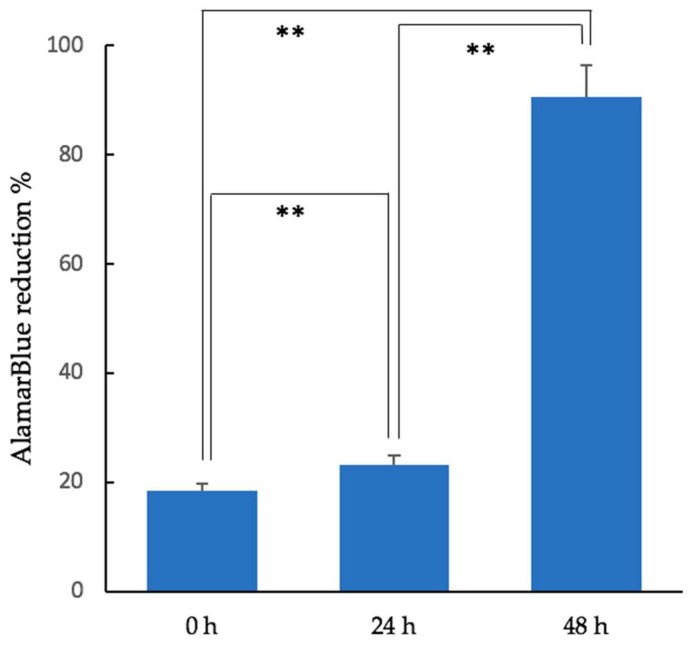
Results of the AlamarBlue assay (fluorescence excitation wavelength: 570 nm; fluorescence emission wavelength: 600 nm) of MC3T3-E1 cells in the cellular cubic composite over 48 h (unpaired *t*-test, ** *p* < 0.01; Error bars represent the standard deviation).

The cell proliferation rate in the 3D-HA/PDLLA cellular cubic composites increased over 2 days of culturing. Cell proliferation demonstrated a dramatic increase by 48 h. The AlamarBlue test results are shown in Figure 2.

### 3.2. Gene Expression of Osteogenic Markers

**Figure 3:**
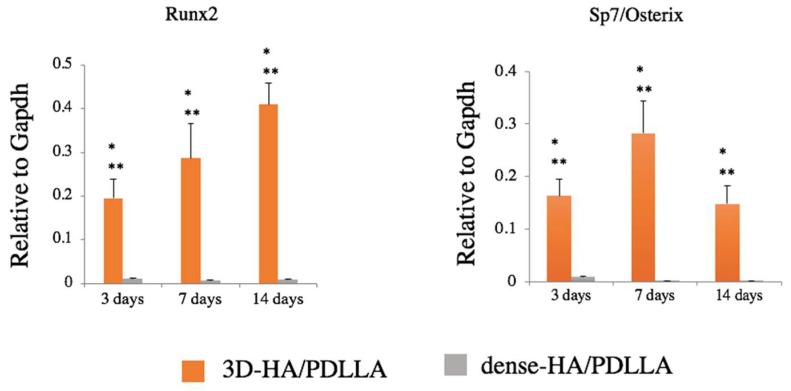
Gene expression of osteogenic markers Runx2 and Sp7/Osterix. Expression was significantly higher in the 3D-HA/PDLLA group (one-factor ANOVA test, * *p* < 0.0001; Tukey–Kramer test, ** *p* < 0.01). n = 3 per group. Error bars represent the standard deviation.

The level of Runx2 expression in the 3D-HA/PDLLA groups increased during osteogenic differentiation and was significantly higher than in the dense-HA/PDLLA groups on days 3, 7, and 14 (*p* < 0.05). Furthermore, the levels of Sp7 (also known as Osterix) were upregulated significantly over time in the 3D-HA/PDLLA group (*p* < 0.05), with the highest levels of expression on day 7 and the lowest on day 14. (Figure 3).

### 3.3. In Vivo Experiments, Histomorphology, and Micro-CT Evaluation

**Figure 4:**
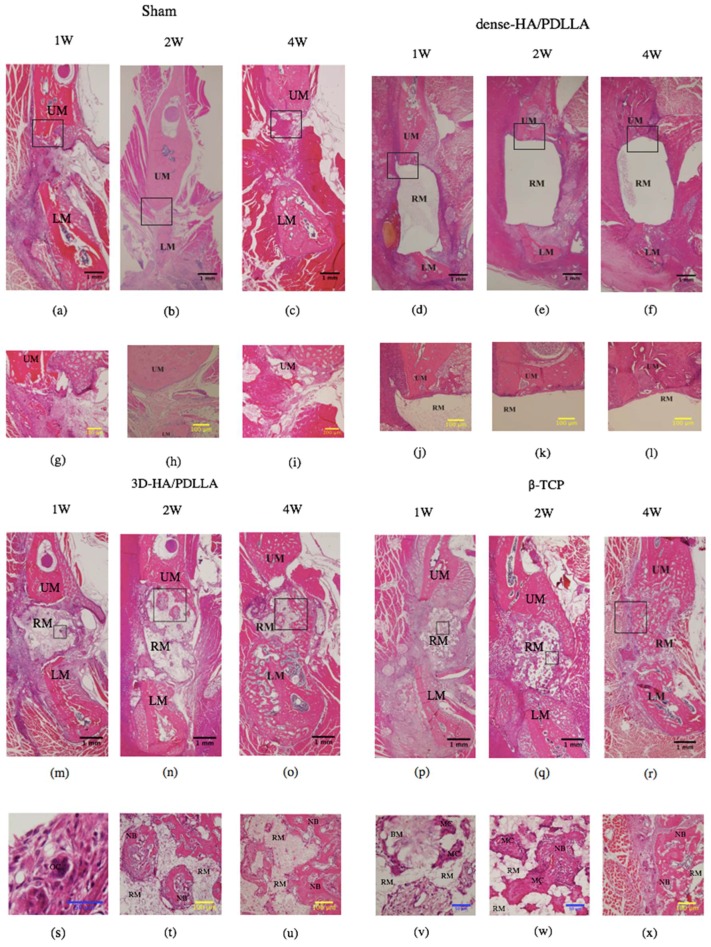
Hematoxylin and eosin staining of decalcified sections. Osteocyte-like cells are seen in the pores in the 3D-HA/PDLLA. The levels of bone formation are nearly identical in the 3D-HA/PDLLA and β-TCP groups. The blue arrowhead points to an osteoclastic-like cell in the pore of 3D-HA/PDLLA, and the black arrowhead to new bone formed in the pores of 3D-HA/DPLLA and beta-tricalcium phosphate (β-TCP). UM: upper mandible; LM: lower mandible; RM: residual material; OC: osteoclast-like cell; NB: newly formed bone; MC: multinucleated cell; BM: bone marrow-like tissue. (**a**–**c**,**g**–**i**) show the sham group; (**d**–**f**,**j**–**l**) show the dense-HA/PDLLA group; (**m**–**o**,**s**–**u**) show the 3D-HA/PDLLA group; (**p**–**r**,**v**–**x**) show the β-TCP group; (**a**,**d**,**g**,**j**,**m**,**p**,**s**,**v**) are at week 1; (**b**,**e**,**h**,**k**,**n**,**q**,**t**,**w**) are at week 2; (**c**,**f**,**i**,**l**,**o**,**r**,**u**,**x**) are at week 4; (**a**–**f**,**m**–**r**) are viewed under 1.25× magnification; (**g**–**l**,**t**,**u**,**x**) are viewed under 10× magnification; (**v**,**w**) are viewed under 20× magnification; (**s**) is viewed under 40× magnification. Scale bars: 1 mm (black), 100 μm (yellow), and 50 μm (blue).

**Figure 5:**
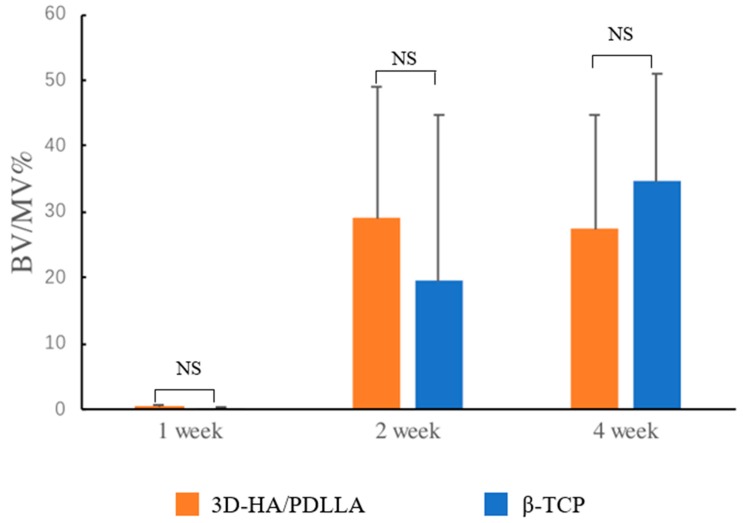
The bone volume (BV) to residual material volume (MV; BV/MV%) was calculated as follows: (area of ectopic bone under 20× magnification/area of residual material under 20× magnification) × 100%. The amount of newly formed bone in the 3D-HA/PDLLA and β-TCP groups was almost identical throughout the test period. The difference in BV/MV% between these two groups was not significant (Kruskal–Wallis H test, *p* > 0.05). n = 3 per group. NS: not significant. Error bars represent the standard deviation.

Histomorphological analysis was performed via H&E staining to assess bone regeneration at 1, 2, and 4 weeks after implantation (Figure 4). No obviously inflammatory reaction was observed by our researchers at any time point in any group (we deemed that without intense inflammatory cell infiltration in H&E staining under 40× magnification, there was no obvious inflammation reaction). There was no obvious bone regeneration in the sham or dense-HA/PDLLA group during follow-up. The border between the biomaterial and bone was clear in the dense-HA/PDLLA group. At low magnification, the 3D-HA/PDLLA and β-TCP groups demonstrated an almost identical level of bone formation (*p* > 0.05; Figure 5). The 3D-HA/PDLLA group showed earlier hydrolysis and absorption than the β-TCP group during the four-week period. Moreover, at high magnification, mild regeneration of bone, cell, and tissue was observed in the pores of the 3D-HA/PDLLA and β-TCP biomaterials at 2 weeks after implantation. During week 4, an abundance of newly formed bone tissue was seen inside the materials and along the boundary between the composite and mandible. Mature ectopic bone formation was not observed in the 3D-HA/PDLLA group nor in the β-TCP group, and the materials were not completely degraded.

**Figure 6:**
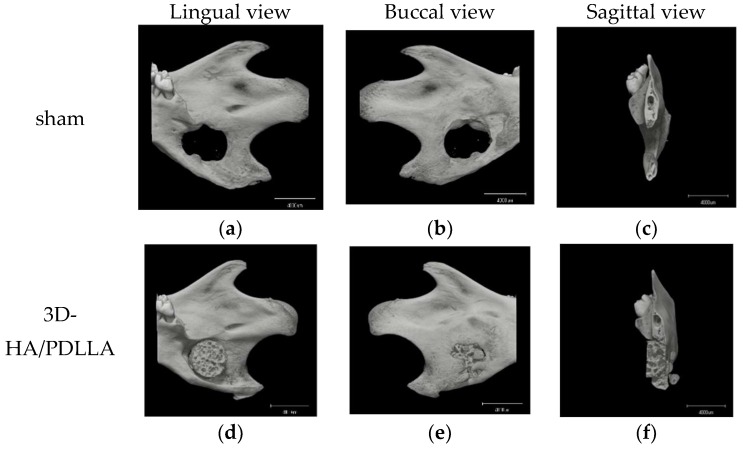

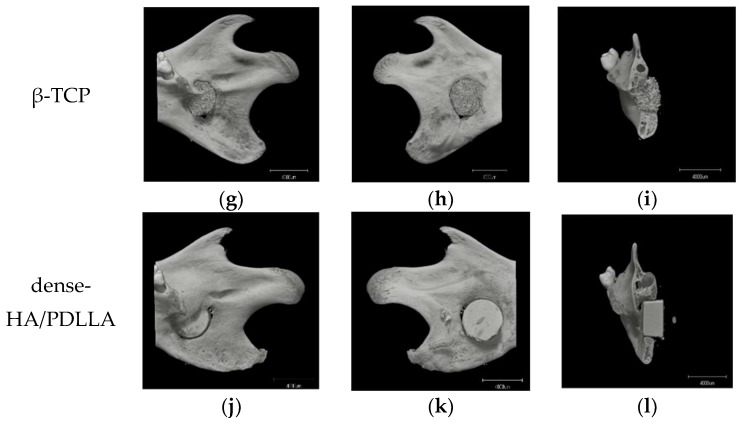
Micro-CT results of the critical bone defect model at 4 weeks after implantation. No obvious bone formation is seen in the sham group. In the 3D-HA/PDLLA and β-TCP groups, some parts of the border between composites and bone cannot be clearly discerned such as (**f**,**i**), unlike in the dense-HA/PDLLA group. Scale bar: 4000 µm.

Micro-CT was performed to analyze osteoregeneration in 3D perspective. Additionally, reconstructed 3D stereoscopic pictures of osteoregeneration were obtained and analyzed. Figure 6 shows the temporal radiographic changes. At the four-week follow-up, some parts of the borders between the biomaterial and mandible became difficult to discern, such as the lower border of 3D-HA/PDLLA (Figure 6f) and the upper border between β-TCP and host bone (Figure 6i), indicating slight integration of the 3D-HA/PDLLA and β-TCP biomaterial with host bone. By contrast, the borders between the materials and host bone could still be clearly seen at 4 weeks in the dense-HA/PDLLA group.

## 4. Discussion

The ideal bone substitute should have good osteoconductivity and/or osteoinductivity, with a controllable degradation rate, be replaced gradually by host bone, and exhibit good biocompatibility, low toxicity, and no induction of inflammation. In addition, due to the 3D complexity of craniofacial bone, malleability and plasticity are also necessary properties. Several studies have described composites of bioactive calcium phosphate and bioabsorbable polymers [17,18,19]. However, because of their mechanical weakness, these materials cannot be used for bone fixation. Ideally, degradable bone substitutes should act as bone fillers that initially fill gaps with slightly higher strength than host bone and retain this strength until bone union [20].

Based on these requirements, biodegradable bone graft substitute made from polymers or copolymers, including polyglycolic acid and poly (l-lactide-co-glycolic acid), have been created and developed for tissue engineering [21,22,23]. Previous reports demonstrated that the osteoconductivity of biopolymers could be enhanced by the addition of HA [24,25]. Recent clinical studies have shown that optimal osteosynthesis fixation devices systems for maxillofacial skeletal surgery could be manufactured with u-HA and poly-l-lactide (HA/PLLA); These composites exhibited satisfactory clinical features during maxillofacial skeletal surgery in terms of bioactive osteoconductivity and bioresorbability (e.g., for orbital wall fracture reconstruction [26,27,28], mandibular reconstructive surgery [29,30], and midfacial trauma osteosynthesis [31]). More recently, bioresorbable composites with improved biocompatible properties made of high-strength HA and PDLLA have been developed for use as grafting biomaterials in the reconstruction of boney defects [32]. In the current study, we used a PDLLA composite with a porous structure u-HA as a bone biomaterial. Our results indicate the improved bone regenerative capability and bonding of this material, as well as its ideal bioactive osteoconductivity, bioresorbability, and 3D shapability. It is therefore appropriate for use in the anatomically and biomechanically complex maxillofacial region.

The properties of this porous biomaterial are very similar to those of cancellous bone. Its macroporous structure allow fibrovascular tissue to easily invade the deeper pore areas [33]. According to previous reports, 50-μm wide pores are sufficient for osteoconduction. However, for superior mechanical strength and osteoconductivity, the optimal pore size is 150–500 μm [34]. The material used in this should satisfy the optimal conditions for osteoconduction. The AlamarBlue assay illustrated the good supportive properties of this composite in cell proliferation (Figure 2). Moreover, as shown in the qRT-PCR analysis, the expression levels of the osteogenic genes Runx2 and Osterix were higher in the 3D-HA/PDLLA group than in the dense-HA/PDLLA group at 3, 7, and 14 days (Figure 3). This result reinforces the finding that biomaterials can profoundly affect osteogenesis during the post-implantation period, promoting cell survival and tissue growth in porous biomaterials.

The gene expression level of Runx2 increased significantly from day 3 to 14 in the 3D-HA/PDLLA group. Runx2 induces the secretion of several factors during chondrocyte differentiation, controls the differentiation of mesenchymal stem cells into osteoblasts, activates COL1 expression, and participates in vessel formation [35,36]. Runx2 influences bone formation and also regulates bone resorption [37]. In the 3D-HA/PDLLA group, total Runx2 RNA expression was significantly upregulated throughout the follow-up period, suggesting that 3D-HA/PDLLA contributes to the remodeling of bone resorption, as well as to bone formation.

Osterix is an essential factor for the condensation of mesenchymal stem cell and the initiation of chondrocyte differentiation. In humans, the mutation of the Osterix gene causes severe chondrodysplasia [38,39]. In Osterix conditional knock-out mice, chondrogenesis during ossification is severely impaired [40]. Our qRT-PCR results showed that the expression of the Osterix gene was significantly higher in the 3D-HA/PDLLA group and peaked on day 7, however, in the dense-HA/PDLLA group, expression remained at a low level. According to Nishimura et al. [41], Osterix is an essential factor for bone tissues that replace cartilage tissues during late-stage endochondral ossification. Importantly, at the hypertrophic stage, a lack of Osterix may hinder endochondral ossification. Furthermore, because Osterix is the downstream transcription factor of Runx2/3, it may regulate the release of matrix metallopeptidase 13 (MMP13) in cartilage during matrix degradation and calcification. This enzyme plays a key role in the regulation of matrix degradation during endochondral ossification [42]. In our study, Osterix expression appears to be erratic, which may be due to the short cell culture period. Therefore, we hypothesize that, with a longer culture time, more consistent results would be obtained.

For the in vivo experiments, we implanted three types of materials into rat mandibles and harvested the samples after 1, 2, and 4 weeks. In the 3D-HA/PDLLA group, the boundary between the biomaterial and mandibular bone was not clear after 4 weeks. Moreover, in the histological assay, newly formed bone cells and tissue were observed in pores in the 3D-HA/PDLLA and β-TCP groups. From 2 to 4 weeks, we also found an increase in new bone formation. Although bone formation characteristics differed between the 3D-HA/PDLLA and β-TCP groups, they showed similar overall bone formation amounts (Figure 4, Figure 5 and Figure 6). In a 1-year follow-up animal study, Akagi et al. [43] reported that the remodeling process with β-TCP likely involved intramembrane ossification. Their 3D-HA/PDLLA group showed fibrous tissue infiltration that markedly exceeded that in their β-TCP group, wherein gradual calcified was seen. This pattern of bone formation suggested endochondral ossification, as also observed by Kondo et al. [44]. It was not immediately clear why the mechanism of ossification in 3D-HA/PDLLA was so similar to that of endochondral ossification. Regardless, in this study, the 3D-HA/PDLLA biomaterial showed very good biocompatibility and osteoconductivity.

In the in vitro study, cell proliferation and differentiation were clearly observed. However, in vivo bone formation took longer than expected, possibly due to micro-environmental differences between the in vivo and ex vivo systems. In vitro, there are sufficient levels of essential elements or nutrients for cell proliferation and differentiation (e.g., essential mineral elements, essential/nonessential amino acids, and basic cell growth factors). In addition, bone regeneration is a complex process that could be affected by several external factors in live animals, and it is impossible to simulate all of these conditions ex vivo. Nonetheless, the results of the in vitro study serve as an important reference.

Horowitz et al. [45] reported positive results with clinical application of β-TCP. However, β-TCP cannot be remolded easily to replace the defect site. In contrast, porous 3D-HA/PDLLA is neither too hard nor too brittle; therefore, it can easily be shaped to fit any bone defect and can be modified using scissors, a scalpel, or thermal methods. This is useful in the context of irregular bone reformation in maxillofacial surgery. According to Hasegawa et al. [6,11], porous 3D-HA/PDLLA biomaterial resorbs much more rapidly than HA or β-TCP. This is very important for bone formation, where Chazono et al. [46] previously reported that bone formation occurred after biomaterial resorption. Therefore, infiltration of osteoclast-like cells plays a role in biomaterial resorption and osteogenesis (osteoclast-like cells indicated by the blue arrowhead in Figure 4s). Additionally, more PDLLA is degraded by hydrolysis; therefore, 3D-HA/PDLLA biomaterial is likely to be useful for patients with osteopetrosis and diabetes [47].

In our in vitro and in vivo experiments, the 3D-HA/PDLLA composite showed a good bioactivity, biocompatibility, and osteoconductivity. However, due to the limitations of our study, we could not measure the mature bone formation or the complete degradation of 3D-HA/PDLLA material and β-TCP. The specific mechanism allowing 3D-HA/PDLLA to function as a bone regeneration scaffold in osteogenesis and cell proliferation/differentiation also remains unknown. In future studies, we will further enhance the capability of this material in promoting bone regeneration by loading it with human mesenchymal stem cells and investigate the performance of 3D-HA/PDLLA as a scaffold in maxillofacial surgery.

## 5. Conclusions

Our cell proliferation, qRT-PCR, and in vivo histological results suggest that 3D-HA/PDLLA will serve as an excellent bioresorbable biomaterial for clinical applications.

## Figures and Tables

**Table 1:** Primers used in Reverse Transcription Quantitative Real-Time Polymerase Chain Reaction (qRT-PCR).

Target Gene (NCBI Accession Number)	Sequence (5′–3′)	Amplicon Size (bp)
Runx2 [12,13] (NM_001278478.1)	F: CCAGATGGGACTGTGGTTACC R: ACTTGGTGCAGAGTTCAGGG	381bp
Sp7/Osterix [14,15] (NM_001300837.1)	F: CTGGGGAAAGGAGGCACAAAGAAG R: GGGTTAAGGGGAGCAAAGTCAGAT	200bp
Gapdh [16] (NM_001289745.2)	F: ACCACAGTCCATGCCATCAC R: TCCACCACCCTGTTGCTGTA	452bp
NCBI, National Center for Biotechnology Information; F: forward primer; R: reverse primer. Annealing temperature was set at 50 °C for all primers, according to the manufacture’s protocol.		
